# Physiological and developmental traits associated with the grain yield of winter wheat as affected by phosphorus fertilizer management

**DOI:** 10.1038/s41598-019-53000-z

**Published:** 2019-11-12

**Authors:** Xiu-Xiu Chen, Wei Zhang, Xiao-Yuan Liang, Yu-Min Liu, Shi-Jie Xu, Qing-Yue Zhao, Yun-Fei Du, Ling Zhang, Xin-Ping Chen, Chun-Qin Zou

**Affiliations:** 1 College of Resources and Environmental Sciences, National Academy of Agriculture Green Development, Key Laboratory of Plant-Soil Interactions, Ministry of Education, China Agricultural University, 100193 Beijing, China; 2 College of Resources and Environment, Southwest University, Chongqing, 400716 China

**Keywords:** Plant physiology, Fertilization

## Abstract

Although researchers have determined that attaining high grain yields of winter wheat depends on the spike number and the shoot biomass, a quantitative understanding of how phosphorus (P) nutrition affects spike formation, leaf expansion and photosynthesis is still lacking. A 3-year field experiment with wheat with six P application rates (0, 25, 50, 100, 200, and 400 kg P ha^−1^) was conducted to investigate this issue. Stem development and mortality, photosynthetic parameters, dry matter accumulation, and P concentration in whole shoots and in single tillers were studied at key growth stages for this purpose. The results indicated that spike number contributed the most to grain yield of all the yield components in a high-yielding (>8 t/ha) winter wheat system. The main stem (MS) contributed 79% to the spike number and tiller 1 (T1) contributed 21%. The 2.7 g kg^−1^ tiller P concentration associated with 15 mg kg^−1^ soil Olsen-P at anthesis stage led to the maximal rate of productive T1s (64%). The critical shoot P concentration that resulted in an adequate product of Pn and LAI was identified as 2.1 g kg^−1^. The thresholds of shoot P concentration that led to the maximum productive ability of T1 and optimal canopy photosynthetic capacity at anthesis were very similar. In conclusion, the thresholds of soil available P and shoot P concentration in whole plants and in single organs (individual tillers) were established for optimal spike formation, canopy photosynthetic capacity, and dry matter accumulation. These thresholds could be useful in achieving high grain yields while avoiding excessive P fertilization.

## Introduction

Wheat (*Triticum aestivum* L.) is a major cereal crop for human consumption and livestock feed^1^. China as the largest wheat producer and consumer in the world has the greatest cultivated area and annual production of wheat^2,3^. However, greater grain yield is still needed to satisfy the increasing population^4^. How to obtain and maintain a sufficient grain yield of wheat is a big challenge.

In the case of winter wheat, obtaining high grain yield depends on the development of sufficient numbers of fertile spikes and sufficient shoot biomass. On the North China Plain (NCP), an important wheat-producing region in China, increasing spike number has been considered the primary way to increase the grain yield of winter wheat^5^. Yield reductions caused by an insufficient number of spikes cannot be compensated for by increases in other yield components^6,7^.

The spike number is determined by fertile tiller initiation in wheat. Only half of the total tillers produce productive tillers that eventually form spikes^8^. The non-fertile tillers that develop in spring may compete for water or nutrients with productive tillers^9,10^. Furthermore, high tiller mortality caused by non-fertile tillers usually contributes to low yield in wheat^11^. Improving productive tillers during wheat growth is vital for achieving high grain yield and reducing tiller mortality. Grain yield has been indirectly associated with tiller dry weight (DW) in winter wheat. It was proven that dry matter accumulation in the main stem (MS) of wheat shows a significant correlation with grain yield^12^. High assimilation ability immediately before anthesis increased the fertility of tiller flowers and the number of grains^13^. Generally, wheat crops with appropriate tillering numbers can develop good spikes through a balanced DW distribution between the productive and unproductive tillers, which is essential to ensuring yield production^14^.

Regulating tiller development at key growth stages of wheat is important in maintaining the balance between tiller mortality and production^15^. Previous studies reported that the application of phosphorus (P) and nitrogen (N) fertilizers affected tiller emergence in wheat^16,17^. Phosphorus levels always greatly affect crop yield^18^. The rate of P application affected the amount and survival of wheat tillers^19^. The emergence of wheat tillers increased linearly with soil P levels, which was mainly attributed to the increased emergence of secondary tillers^14^. Shoot P concentration could also affect tiller emergence and especially the emergence of the first tiller (tiller 1 or T1)^20,21^. These results indicate that P nutrition in wheat plants plays an important role in generating fertile tillers. However, the critical requirement for P nutrition in shoots and especially in individual tillers which facilitate fertile spike formation by balancing the tiller DW at key stages, while also avoiding excessive P addition, remain unclear.

Although researchers have determined that increasing the yield of modern wheat varieties also depends on increasing the harvest index (HI), i.e., the fraction of the aboveground biomass represented by grain, the HI of wheat has not been significantly increased since the early 1990s^22–24^. It follows that increases in yield will likely depend on increases in aboveground biomass. Close correlations were observed between yield and shoot biomass at the anthesis, post-anthesis, and maturity stages of wheat, and differences in biomass among different yield categories began at anthesis^5^. Previous studies also reported that a large proportion of grain yield was derived from shoot dry matter that was produced before anthesis and translocated to the grain during the grain filling stage^25,26^. This suggests that increasing total shoot biomass, especially at anthesis, is fundamental to achieving high yield. Phosphorus application increased the straw biomass and yield in wheat ^19, 27^, whereas P deficiency reduced the shoot growth of winter wheat^28^.

Photosynthesis is the ultimate source of biomass in plants^29,30^. The biomass of crops is also associated with the leaf area index (LAI) due to its effect on light interception^31^. However, P deficiency restrains wheat growth by decreasing the leaf expansion rate and assimilation^26,27,32,33^. In contrast, appropriate phosphate fertilizer application can simultaneously increase the photosynthesis and leaf area of wheat^28,34^.

We hypothesize that both the spike formation and photosynthesis characteristics of winter wheat can be optimized to realize high yields by regulating P nutrition from the soil to the plants at crucial stages. It remains unclear how P nutrition regulates the tiller development dynamic and the subsequent spike formation and how to regulate P nutrition to coordinate spike formation and biomass accumulation to achieve maximum grain yields in wheat while avoiding excessive P fertilization.

The aims of this study in winter wheat were to (1) clarify the critical P nutrition level that satisfies the requirements of individual tiller development and subsequent spike formation, (2) determine the responses of LAI, Pn, and the product of Pn and LAI to the P concentrations in the soil and the shoot that are associated with shoot biomass, and (3) quantify the P concentration in shoot which is combined the spike quantity with shoot biomass to achieve the maximum grain yield of wheat.

## Results

### Grain yield, biomass, yield components, harvest index, and P content at maturity

Across the three cropping years, the grain yield and shoot biomass of winter wheat at maturity increased with P application rate and plateaued when grain yield achieved 8–9 Mg ha^−1^ (Table 1). The harvest index (grain biomass/aboveground wheat biomass) ranged between 0.4 and 0.5, irrespective of the P application rate. Phosphorus concentration and accumulation in grain and straw continued to increase or plateaued after increasing as P application increased.

**Table 1:** Grain yield (14% water content), shoot (grain and straw) biomass, yield components, harvest index (HI), and P concentration and accumulation in grain and shoots at maturity as affected by P application rate in three cropping years.

Harvest year	P application rate (kg P ha^−1^)	Yield (Mg ha^−1^)	Biomass (Mg ha^−1^)	Number of spikes m^−2^	Number of grains spike^−1^	TKW (g)	HI	P concentration (g kg^−1^)		P accumulation (kg ha^−1^)	
								Grain	Straw	Grain	Straw
2015	0	4.90b	9.38c	427d	24.3b	39.5a	0.45 cd	2.13d	0.16c	9.01d	0.81b
	25	8.30a	14.3b	625c	30.2a	39.0a	0.50a	2.87c	0.27c	20.5c	1.93b
	50	8.67a	15.5ab	708bc	31.3a	35.6b	0.48ab	3.21b	0.46b	24.0b	3.84a
	100	8.33a	16.5a	764ab	32.2a	33.4c	0.44d	3.88a	0.43b	27.8a	4.02a
	200	8.26a	15.5ab	795ab	30.4a	32.0c	0.46bcd	4.19a	0.54ab	29.8a	4.55a
	400	8.50a	15.7ab	871a	33.1a	32.5c	0.47bc	4.21a	0.60a	30.7a	4.98a
2016	0	2.58d	5.12e	462c	20.5c	36.5c	0.41b	2.73d	0.19c	6.02d	0.57c
	25	5.25c	8.39d	482bc	30.4b	43.1a	0.54a	2.96 cd	0.18c	13.4c	0.70c
	50	6.72b	11.5c	526ab	35.3a	42.8a	0.50a	3.28bc	0.27b	19.0b	1.58b
	100	7.69a	12.7b	566a	35.1a	41.1b	0.52a	3.70a	0.30b	24.4a	1.87b
	200	7.86a	12.9b	580a	35.0a	40.8b	0.52a	3.61ab	0.31b	24.4a	1.89b
	400	8.30a	14.5a	581a	35.3a	42.0ab	0.49a	3.72a	0.39a	26.6a	2.88a
2017	0	4.35c	7.97c	481c	28.2c	36.8ab	0.47b	2.43c	0.21e	9.23d	0.92e
	25	7.82b	13.6b	616b	38.6a	37.9a	0.49a	2.70c	0.29de	18.1c	2.00d
	50	8.68ab	15.3ab	829a	35.6ab	35.8abc	0.48ab	3.23b	0.37 cd	24.1b	2.90 cd
	100	8.23ab	14.3b	886a	35.8ab	33.4c	0.49a	3.34b	0.49b	23.7b	3.55bc
	200	9.21ab	16.8a	878a	31.9bc	33.1c	0.47ab	3.53ab	0.46bc	27.8ab	4.12b
	400	9.57a	17.1a	876a	33.5b	34.5bc	0.48ab	3.69a	0.63a	30.3a	5.64a
**Source of variation**											
P application rate (P)	***	***	***	***	***	**	***	***	***	***	
Harvest year (Y)	***	***	***	***	***	***	**	***	***	***	
P*Y	**	**	***	***	***	**	***	**	ns	**	

TKW means thousand-kernel weight. Values are means of four replications. Within each year, means followed by same letters are not significantly different (P < 0.05). ANOVA results are shown at the bottom: ** and *** indicate significance at P < 0.01 and < 0.001, respectively, and “ns” indicates no significant difference.

The number of spikes m^−2^ at harvest increased with the P application rate from 0 to 100 kg ha^−1^ in 2015 and from 0 to 50 kg ha^−1^ in 2016 and 2017 and then plateaued. The number of spikes was greater in 2015 and 2017 than in 2016. The number of grains spike^−1^ increased as the P application rate increased from 0 to 25 kg ha^−1^ in 2015 and 2017 and from 0 to 50 kg ha^−1^ in 2016 and then plateaued. The thousand-kernel weight (TKW) began to decrease when the P application rate increased to 25 kg ha^−1^ in 2015 and 2016 and to 50 kg ha^−1^ in 2017. The thousand-kernel weight tended to be greater in 2016 than in the other 2 years (Table 1). All these parameters were significantly different between the cropping years. In addition, there were significant effects of the P rate and crop year on all the parameters except grain P accumulation (Table 1). The spike number explained 50% of the variation in yield. The number of grains per spike and TKW explained only 13% and 3%, respectively, of the variation in yield (Supplementary Table 1).

### Individual stem P concentration regulates spike formation by balancing stem dry weight

**Figure 1:**
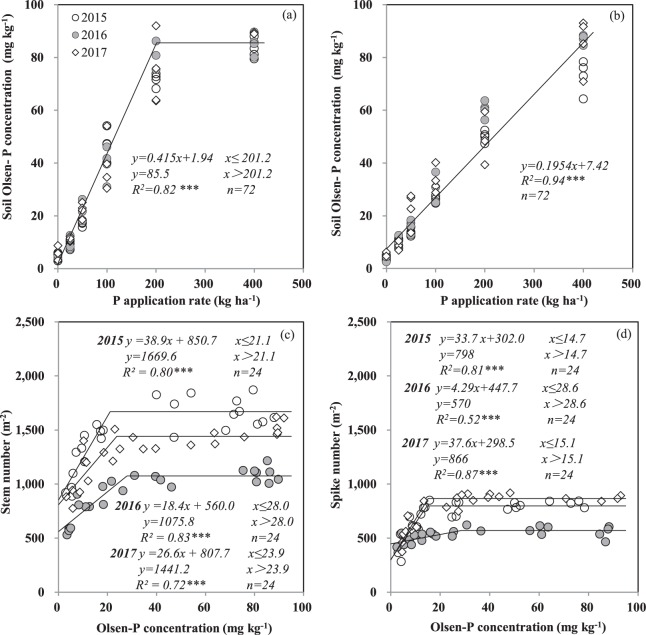
Olsen-P concentration in soil as affected by P application rate and its correlation with stem number at stem elongation (**a–c**) and spike number at anthesis (**b–d**) stages of winter wheat, respectively. Each data point is from one plot in 1 year. *** indicates significant difference at *P* < 0.001.

For all 3 years, the soil Olsen-P concentration showed a linear-plateau relationship with the P application rate at the stem elongation stage (Fig. 1a). The stem number of wheat first increased with soil Olsen-P concentration and then plateaued (Fig. 1c). At the anthesis stage, the soil Olsen-P concentration increased linearly with the P application rate (Fig. 1b). The spike number at the anthesis stage increased linearly with the soil Olsen-P concentration until a plateau of 15 mg kg^−1^ was reached in 2015 and 2017 (Fig. 1d). The critical level of the soil Olsen-P concentration was higher in 2016 than in 2015 and 2017.

**Figure 2:**
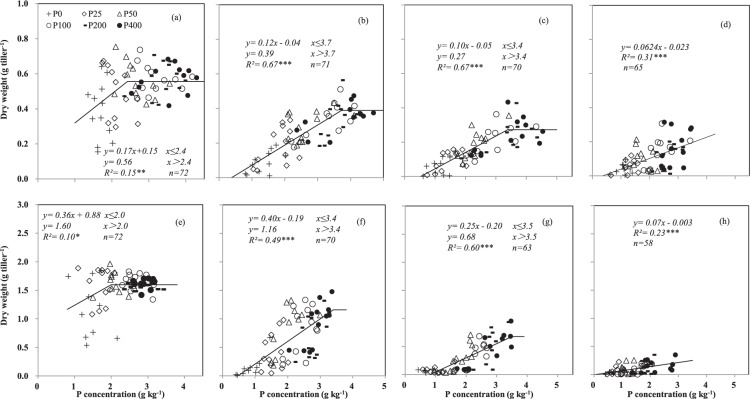
The relationship between the dry weight and P concentration in the main stem, tiller 1, tiller 2, and other tillers of wheat at the stem elongation stage (**a–d**), respectively) and at the anthesis stage (**e–h**), respectively) across P application rates and cropping years. Each data point from one plot in 1 year. The sample size which is not enough 72 means no tiller (e.g. T1, T2, or OT) was found in harvested plants in some specific experiment plots. *, ** and *** indicate significance at P < 0.05, P < 0.01 and P < 0.001, respectively.

The DW of all stems except for other tillers increased with the P concentration in the respective stem and then plateaued at both the stem elongation and anthesis stages (Fig. 2). At the stem elongation stage across the 3 years, the DW values of main stem, tiller 1, and tiller 2 were highest at stem P concentrations of 2.4, 3.7, and 3.4 g kg^−1^, respectively (Fig. 2a–c). At anthesis, the DW values of the main stem, tiller 1, and tiller 2 were greatest at P concentrations of 2.0, 3.4, and 3.5 g kg^−1^, respectively (Fig. 2e–g). The dry weight of other tillers at stem elongation and anthesis increased steadily with stem P concentration (Fig. 2d–h).

**Figure 3:**
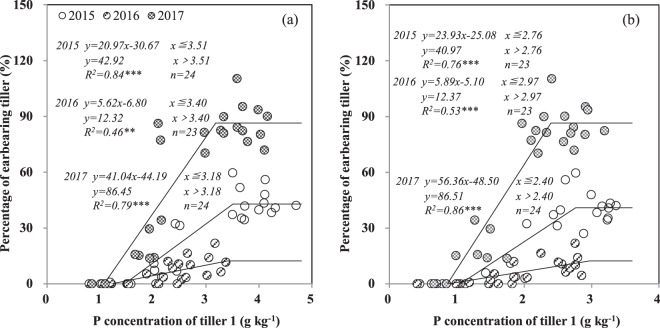
The relationship between P concentration of T1 at stem elongation stage (**a**) and anthesis stage (**b**) and percentage of earbearing tiller in 2015, 2016 and 2017. The sample size which is not enough 24 means no tiller 1 was found in harvested plants in some specific experiment plots. **, *** indicates that regressions are significant at *P* < 0.01 and *P* < 0.001.

The percentage of earbearing tillers on the MS in three years was improved by P addition. The percentage of earbearing T1 tillers increased with the P application rate from 0 to 50 kg ha^−1^ in 2015 and 2017 and from 0 to 100 kg ha^−1^ in 2016 and then plateaued (Table 2). The contribution rate of the MS to spikes was decreased, while the contribution of T1 increased, as P application rates increased, except that of the MS in 2016 (Table 2). The percentage of earbearing T1 tillers was increased by the tiller P concentration and then plateaued at both the stem elongation and anthesis stages (Fig. 3). The critical P concentration in T1 for the percentage of earbearing tillers was 3.4 mg kg^−1^ at the stem elongation stage (Fig. 3a) and 2.7 mg kg^−1^ at the anthesis stage (Fig. 3b).

**Table 2:** The percentage of earbearing tiller (%) and the contribution rate to spikes (%) of main stem (MS) and tiller 1 (T1) of per wheat plant as affected by P application in 3 years.

Year	P level (kg P ha^−1^)	Percentage of earbearing tiller		Contribution rate to spikes	
		Main stem	Tiller 1	Main stem	Tiller 1
2015	0	65b	0.0b	99.3a	0.7c
	25	100a	5.8b	91.2a	8.8c
	50	100a	37.2a	68.7bc	31.3ab
	100	100a	45.6a	69.3bc	30.7ab
	200	100a	37.4a	72.8b	27.2b
	400	100a	42.1a	62.2c	37.8a
2016	0	88b	0.0b	100a	0.0c
	25	91b	0.2b	99.8a	0.2c
	50	98a	1.9b	98.2ab	1.8bc
	100	100a	10.7a	92.7bc	7.3ab
	200	100a	11.7a	89.9c	10.1a
	400	99a	8.41a	94.3abc	5.7abc
2017	0	95b	7.3c	93.6a	6.4c
	25	100a	23.3b	81.5b	18.5b
	50	100a	78.8a	56.0c	44.0a
	100	100a	87.9a	53.2c	46.8a
	200	100a	89.4a	53.0c	47.0a
	400	100a	82.0a	55.1c	44.9a
**Source of variation**					
P application rate (P)	***	***	***	***	
Year (Y)		**	***	***	***
P*Y		***	***	***	***

Values are means of four replications. Within every year, means followed by different letters are significantly different (P < 0.05). ANOVA results are shown at the bottom: **, and *** indicate significance at P < 0.01, and P < 0.001 levels, respectively.

### Relationships between yield, shoot biomass, canopy photosynthetic capacity, shoot P concentration, and soil Olsen-P concentration

**Figure 4:**
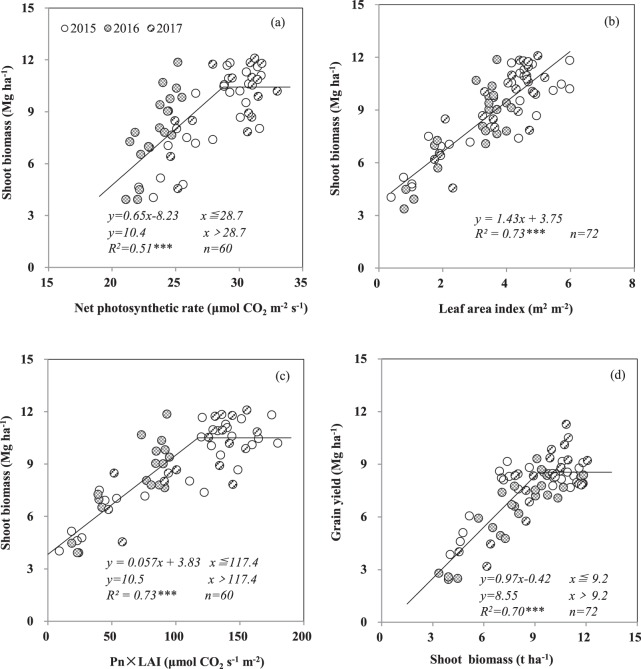
Relationships between shoot biomass at anthesis and net photosynthetic rate (**a**), leaf area index (**b**), product of net photosynthetic rate and leaf area index (**c**), and grain yield (**d**) across 3 years. Each data point comes from one plot in 1 year. *** indicates significance at *P* < 0.001.

The shoot biomass of winter wheat at anthesis showed a linear-plateau relationship with the net photosynthetic rate (Pn) (Fig. 4a) and a linear relationship with the leaf area index (LAI) (Fig. 4b). The relationship between shoot biomass and the value of Pn × LAI showed similar a tendency as the relationship between shoot biomass and Pn (Fig. 4c). The grain yield of winter wheat increased with shoot biomass at anthesis and then plateaued when the shoot biomass reached 9.2 Mg ha^−1^ (Fig. 4d). An LAI of 3.8 m^2^ m^−2^ and a Pn of 26.8 μmol CO_2_ m^−2^ s^−1^ will satisfy the development needs of shoots to achieve the optimal biomass. The corresponding value of Pn × LAI was 94.2 μmol CO_2_ s^−1^ m^−2^.

**Figure 5:**
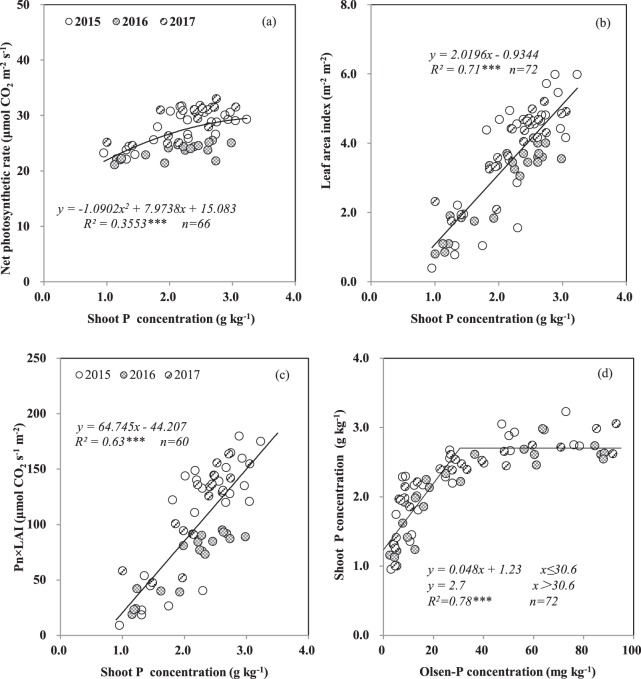
Relationships between net photosynthetic rate and shoot P concentration (**a**), between leaf area index and shoot P concentration (**b**), between the product of net photosynthetic rate and leaf area index and shoot P concentration (**c**), and between shoot P concentration and soil Olsen-P concentration (**d**) at anthesis across three years. Each data point from one plot in 1 year. *** indicate significance at *P* < 0.001.

The values of LAI, Pn, and Pn × LAI were regulated by the shoot P concentration (Fig. 5a–c). The critical shoot P concentration levels for realizing the corresponding Pn (26.8 μmol CO_2_ m^−2^ s^−1^), LAI (3.8 m^2^ m^−2^), and Pn × LAI (94.2 μmol CO_2_ s^−1^ m^−2^) were 2.0, 2.3, and 2.1 g kg^−1^, respectively, for the 3 years. As the available soil P concentration at the anthesis stage increased, the P concentration in the shoots first increased and then plateaued (Fig. 5d).

## Discussion

### Effect of P application on spike formation associated with tiller P nutrition and soil P

The wheat in present study was a high-tillering cultivar that is widely cultivated on the NCP according to the climate and environmental characteristics of this area. The wheat yield of high-tillering cultivars relies on having enough fertile spikes rather than on a large spike size^6,35^. The grain yield of winter wheat in the current study was more determined by the spike number m^−2^ than by the TKW or grain number spike^−1^. The result was also supported by previous findings that spike number is the primary determinant of wheat yield^5,36,37^. Increasing other yield components cannot compensate for the reduction in grain yield associated with inadequate spike number. For instance, the extremely low temperatures in January and February of 2016 (Supplementary Fig. 1b) in the current study resulted in a low spike number that could not be compensated for by increases in grain number spike^−1^ or TKW (Table 1). The inadequate number of spikes may be attributed to the inhibited tiller occurrence and higher death of fertile florets under low temperatures in spring^38^.

In present study, the fertile spikes were resulted from the MS and T1 of wheat. Main stem and T1 contributed 79% and 21%, respectively, to the spike number at harvest across the 3 years. Similar results were found in high-yield systems in the UK and Germany, in which harvest spikes also resulted from MS and somewhat from T1^39,40^. An adequate spike number m^−2^ resulted from balanced development among the MS, T1, T2, and other tillers at key stages. The pre-winter stem number did not increase a lot with P application (Supplementary Fig. 3), which may have been caused by the slow plant growth in the winter, although the early tillering of wheat resulted in increased grain yield according to Baker and Gallagher^41^. The stem number at the stem elongation stage reached the maximum as the soil Olsen-P increased to 24.3 mg kg^−1^. Unlike pre-winter stem production, however, excessive tiller production at stem elongation stage results in many unproductive tillers^9,42^ and higher tiller mortality^43^. A previous report mentioned the relationship between the tillering rate and the shoot P content of wheat plants^44^. However, to our knowledge, studies about the relationships between individual tiller P concentrations, tiller development, and the spike formation ability of tillers are absent. In the current study, the critical tiller P concentrations for DW of the main stem and T1 were 2.4 and 3.7 g kg^−1^ at the stem elongation stage, respectively. However, a tiller P concentration of 3.4 g kg^−1^ can led to the highest productive percentage of first tillers (65%) at the stem elongation stage, as seen in 2015 and 2017 in this study. A previous study found that 53% of T1 were productive at harvest, which is less than that found in the current study and may have been caused by rainfed growth condition^45^. Although the DW of tiller 2 and other tillers also increased with the P application rate, which agrees with the results of Khan *et al*.^46^, these tillers are mostly non-fertile and contribute to low grain yields. P application should promote the development of the main stem and tiller 1 while controlling the tiller quantity to avoid competition. To our knowledge, this is the first study to establish a critical P concentration at the organ (tiller) level under field conditions.

The termination of wheat tillers occurs between the onset of stem elongation and anthesis^47^. Understanding the tiller P conditions associated with soil P conditions is crucial for avoiding excessive P application to superfluous tillers from the stem elongation to the anthesis stage. In present study, the highest DW of MS reached 1.6 g tiller^−1^ and was affected by the increasing tiller P concentration. In a previous study under pot conditions, the DW of main stem at anthesis stage increased by 2.0–2.5 g stem^−1^ in two different cultivars as the P application rate increased to a critical value^14^. Such a difference in the DW of main stem may be attributed to the better vegetative growth of tillering wheat in pot conditions than in field conditions and to the different wheat cultivar. A higher DW in tillers does not always indicate a higher probability of the tiller being productive. For individual tillers, P concentrations of 3.4 g kg^−1^ at anthesis stage will result in the maximal quality of tiller 1. However, in this study, the 0.89 g tiller^−1^ of tiller DW resulting from 2.7 g kg^−1^ tiller P concentration ensured that 64% of first tillers would be productive; this tiller P concentration was associated with the value of soil Olsen-P concentration (15 mg kg^−1^) that resulted in the maximal fertile spike number. The development of tiller 1 was significantly influenced when the P concentration in shoots was less than 5 g kg^−1^ according to Sato *et al*.^21^. Our results indicate that shoot P content at 2.0 g kg^−1^ led to the highest productive ability in T1 at anthesis (Supplementary Fig. 2b). This shoot P concentration is quite close to the threshold for realizing the best canopy photosynthetic capacity. The results indicated that approximately 1.6 spikes per plant can be expected to achieve high yield under a soil Olsen-P concentration of 15 mg kg^−1^ at anthesis stage. On the NCP, it was reported that 1.5 spikes per plant was found in experimental plots with grain yields of 7–8.5 t ha^−1^ and 2.1 spikes per plant were found in experimental plots with grain yields > 8.5 t/ha^36^. These results were similar to the present results in 2015 (1.4 spikes plant^−1^) and 2017 (1.9 spikes plant^−1^). According to this study, higher soil Olsen-P concentrations (exceeding 15 mg kg^−1^) did not further increase the population of fertile spikes.

### The role of Pn, LAI, and Pn × LAI in achieving high yield were affected by soil and shoot P conditions

Maintaining high yield and efficient production is a constant challenge in intensive agricultural systems. Across the 3-year study, P fertilization resulted in a winter wheat grain yield of 8–9 Mg ha^−1^, which is substantially greater than the average yield (6.6 Mg ha^−1^) for the NCP^48^. The maximal harvest index (HI) of winter wheat in northern China was previously reported to have remained at approximately 0.5 for several decades^49^, a value that was consistent with the current results and that probably cannot be increased. Austin *et al*.^50^ suggested that the HI limit of wheat was 0.62, but since the early 1990s, there has been no systematic increase in HI from values of 0.50 for spring wheat^22,23^ and 0.55 for winter wheat^24^. Increasing shoot biomass is evidently the most practical approach to increasing grain yield in the current wheat production system.

The shoot biomass of wheat at the anthesis stage was determined by Pn and LAI and their product. Insufficient photosynthetic capacity of winter wheat during anthesis stage led to an insufficient shoot biomass for crop yield in the current study. This is consistent with studies conducted in southeastern China that demonstrated the decreased Pn and LAI would reduce grain yield of winter wheat^51,52^. On the other hand, higher Pn and LAI resulting from P fertilizer application did not always result in higher yield. A study conducted in northern Germany also verified that the grain yield of winter wheat increased with the green area index (GAI, the ratio of total surface area of green components of a canopy per area of ground) at different vegetative growth stages, and plateaued at the critical levels of GAI^53^.

In present study, LAI showed a better correlation with shoot biomass than that of Pn. This finding was supported by Jiang *et al*.^54^, who noted that LAI showed a higher coefficient with wheat yield than Pn. According to Zhang *et al*.^55^, the LAI and Pn of maize grown in the field were directly determined by the P concentration in shoots and thereby associated with available soil P conditions, which was consistent with the results of the present study. However, Jiang *et al*.^54^ clarified that the parameter for canopy photosynthetic capacity of Pn × LAI was much more important for wheat yield than the single-leaf photosynthetic capacity. The result was similar to that in this study, in which the relationship between Pn × LAI and shoot biomass showed a coefficient of 0.731. The critical shoot P concentration for realizing the optimal Pn × LAI was identified as 2.1 g kg^−1^ associated with a soil Olsen-P concentration of 18 mg kg^−1^. This level of soil Olsen-P concentration is slightly lower than the previously reported recommendation for the agronomic demands of winter wheat^56^.

### How to manage P application in production

According to the above results, 18 mg kg^−1^ of soil Olsen-P at the anthesis stage can satisfy the requirements for optimized spike formation, canopy photosynthetic capacity, and dry weight accumulation combined with high grain yield. This soil Olsen-P concentration was slightly lower than that found in a previous study, which demonstrated that characterization of the roots responded best at approximately 20 mg kg^−1^ soil Olsen-P concentration at anthesis stage^56^.

In practice, farmers generally believe that “more fertilizer equals more grain yield”, so excessive P fertilizer is usually applied for wheat on the NCP and in other areas^57^. Over-use of P fertilizer not only wastes P resources and pollutes the environment^58^ but also reduces grain quality in factors such as zinc concentration^59^. Therefore, P fertilizer application should be managed to provide enough soil Olsen-P to achieve high grain yield based on the development of both physiological and developmental traits while avoiding excessive P application. The “building-up/maintenance” approach is used in many developed countries and in China according to the soil P availability category^60,61^. There are complex interactions between applied fertilizer P and different soil components; however, in a given soil type, the relationship between P application and changes in soil available P should be established for this “building-up/maintenance” approach^62^. In the current study, the relationships among soil available P, plant P, and plant physiological and developmental traits were further established, and it could be helpful to understanding the mechanisms of yield formation and thereby managing P in a sustainable way in intensive wheat production systems.

## Conclusion

The spike number contributed the most to grain yield among all yield components in this study of high-yielding winter wheat. The main stems and first tillers were main sources of fertile spikes. Tiller density and dry weight need to be optimized through appropriate shoot and individual-tiller P nutrition under the regulation of soil P at vital stages to realize the highest number of productive tillers. The study indicates that approximately 1.6 spikes per plant can be expected to achieve high yield under a critical shoot P concentration of 2.0 g kg^−1^ with 15 mg kg^−1^ soil Olsen-P concentration at the anthesis stage. The thresholds of shoot P concentration that led to the best canopy photosynthetic capacity and the highest productive ability of T1 at anthesis were very similar. Based on the requirement of both physiological and developmental characteristics, 18 mg kg^−1^ soil Olsen-P was sufficient to provide adequate P nutrition for shoot and individual tiller development and for canopy photosynthetic capacity to achieve high yield. In conclusion, the high grain yield of wheat was attributed to having sufficient shoot biomass and spike number, which were regulated by P nutrient transfer from soil to plants.

## Materials and Methods

### Field location

A long-term field experiment was initiated in 2008 in Quzhou (36.9°N, 115.0°E), Hebei Province, China. The present results were obtained from three continuous cropping seasons: 2014–2015, 2015–2016, and 2016–2017 (recorded as 2015, 2016, and 2017, respectively). The average monthly temperature and precipitation data from 2015 to 2017 are provided in Supplementary Fig. 1. The soil was a calcareous alluvial soil (WRB, Fluvisol) with a pH of 8.0 (1:2.5 w/v in water).

### Experimental design

The treatments included six P application rates: 0, 25, 50, 100, 200, and 400 kg P ha^−1^. Phosphorus fertilizer was applied annually to winter wheat and summer maize (1/2 the amount of winter wheat) from 2008 until the present. Phosphorus was applied as calcium superphosphate (7% P). Each treatment was represented by four replicated plots (10 × 7.5 m per plot), and the same plots were used for each P fertilizer treatment in every season for all 3 years. Winter wheat (*Triticum aestivum* L., cv Liangxing 99) was sown at a rate of 5.6 × 10^6^ ha^−1^ in each year. Before sowing, 75 kg ha^−1^ of nitrogen (N) in the form of urea (46% N) and 60 kg ha^−1^ of K_2_O in the form of potassium sulphate were applied to the soil, and another 150 kg ha^−1^ of N in the form of urea (46% N) was applied at the stem elongation stage. In each of the three cropping seasons, 700 m^3^ ha^−1^ of irrigation water was applied at three times: the pre-winter, stem elongation and anthesis stages. No standing water, pest, or weed problems were observed during the experiment. Winter wheat was sown on 8 October 2014, 7 October 2015, and 7 October 2016 and harvested on 7 June 2015, 8 June 2016, and 2 June 2017, respectively.

### Sample collection and analysis

Stems were counted at the seedling, pre-winter, stem elongation, anthesis, and maturity stages in two separate subplots (0.25 m^2^ per subplot) in the central rows of each plot. At the stem elongation and anthesis stages, 30 adjoining and uniformly grown plants were hand collected at the soil surface level. Each plant was separated into the main stem (MS), tiller 1 (T1, tiller in the axil of the first leaf on the MS), tiller 2 (T2, tiller in the axil of the second leaf on the MS), and other tillers (OT) to determine the biomass and P concentration of stems. Plant samples were washed with deionized water, dried to a constant DW at 60–65 °C, and then ground to pass through a 0.5 mm sieve with a stainless-steel grinder for P analysis.

In each plot, 6 soil samples (0–20 cm) were collected in an ‘S’ pattern at the stem elongation and anthesis stages and composited. Soil samples were air dried and then passed through a 2-mm plastic sieve for soil Olsen-P analysis. Shoot samples (the whole aboveground plant) were collected in two 0.125 m^2^ subplots in two adjacent rows at the stem elongation, anthesis, and maturity stages. The shoot samples were immediately washed with tap water and then with deionized water and were dried at 60–65 °C to a constant DW. The dried shoot samples were ground with a stainless steel grinder for P analysis.

At maturity, a 6-m^2^ (2 × 3 m) subplot in the centre of each plot was harvested to determine grain yield. Grain samples were rapidly washed with deionized water^63^ and then dried at 60–65 °C to a constant weight to determine yield (14% water content) and P concentration. The thousand-kernel weight (TKW) was also determined for each plot. For the determination of grain number per spike, the grains were counted on 30 uniform spikes from approximately 0.5 metre adjacent plants per plot.

The photosynthesis rate and LAI were determined at the anthesis stage. A portable non-dispersive CO_2_/H_2_O infrared gas analyser (LC*pro*-SD, ADC BioScientific Ltd. UK) was used to measure the Pn of flag leaves of three selected plants per plot between 9:30–11:30 am on a sunny day. Four and three replications of each treatment were tested in 2015 and in 2016 and 2017 for Pn measurement, respectively. The leaf area index was measured three times using the SunScan canopy analysis system (Delta-T Devices, Cambridge, UK) between 10:00–12:00 am on a sunny day for all plant leaves in each plot.

The soil Olsen-P concentration was measured by the colorimetric method based on extraction with NaHCO_3_^64^. The grain and shoot samples were digested with HNO_3_-H_2_O_2_ in a microwave-accelerated reaction system (CEM, Matthews, NC, USA), and the P concentration in the digested solutions was determined using inductively coupled plasma optical emission spectroscopy (ICP-OES, OPTIMA 3300 DV, Perkin-Elmer, USA). Standard materials IPE126 and IPE568 (WEPAL, Netherlands) were used in the analysis of P in shoot and grain samples, respectively.

### Calculations

The percentage of earbearing tillers of MS plant^−1^ (%) = 100 (when the number of fertile spikes was higher than the number of initial seedlings) or the number of fertile spikes/number of initial seedlings × 100.

The contribution rate of the MS to spikes (%) = 100 (when the number of fertile spikes was lower than the number of initial seedlings) or the number of initial seedlings/number of fertile spikes × 100.

The percentage of earbearing T1 tillers plant^−1^ (%) = (number of fertile spikes-number of initial seedlings)/number of initial seedlings × 100 (the value equals 0 when the number of fertile spikes was lower than the number of initial seedlings).

The contribution rate of T1 to spikes (%) = (the number of fertile spikes-the number of initial seedlings)/the number of fertile spikes × 100 (the value equals 0 when the number of fertile spikes was lower than the number of initial seedlings).

### Statistical analysis

Excel 2010 (Microsoft, USA) was used for calculations. Data from the three cropping years were subjected to two-factor analyses of variance (ANOVAs) to evaluate the effect of P application rate and year on grain yield, the three major yield components, and other variables at the maturity stage. Duncan’s test was used to compare means. Linear regression was used to represent the relationships between soil Olsen-P concentration and P supply at anthesis stage, between shoot biomass and LAI, and so on. Quadratic regression was used to represent the relationships between net photosynthetic rate and shoot P concentration. In the linear-plateau model, the point at which an increase in the independent variable no longer results in an increase in the dependent variable is termed the “critical point” or the “critical rate”. The inclined segment is described by the equation y = ax + b (if x ≤ critical point), and the horizontal segment is described by the equation y = c (if x > critical point), where “a” is the slope of the inclined segment and “b” and “c” are intercepts. SAS software (SAS 8.0, USA) was used to conduct the analyses and to obtain the relevant parameters. The treatments were repeated in the same plots during the experiment with rotation of winter wheat and summer maize. Before analysis, data normality and homogeneity of variance were checked by SPSS 20.0 (*P* > 0.05).

## Supplementary information

Supplementary

## Data Availability

The datasets in the current study could be made available from the corresponding authors upon request.
