# Supplementary material for: Physiological and developmental traits associated with the grain yield of winter wheat as affected by phosphorus fertilizer management
Source: Sci Rep. 2019 Nov 12;9:16580. doi: 10.1038/s41598-019-53000-z (PMC6851383; doi:10.1038/s41598-019-53000-z)
Supplement: Supplementary file 1 — Supplementary [file 41598_2019_53000_MOESM1_ESM.pdf]

Physiological and developmental traits associated with the grain yield of winter wheat as affected by phosphorus fertilizer management

Xiu-Xiu Chen[1], Wei Zhang[2], Xiao-Yuan Liang[1], Yu-Min Liu[1], Shi-Jie Xu[1], Qing-Yue Zhao[1], Yun-Fei Du[1], Ling Zhang[1], Xin-Ping Chen[2], Chun-Qin Zou[1*]

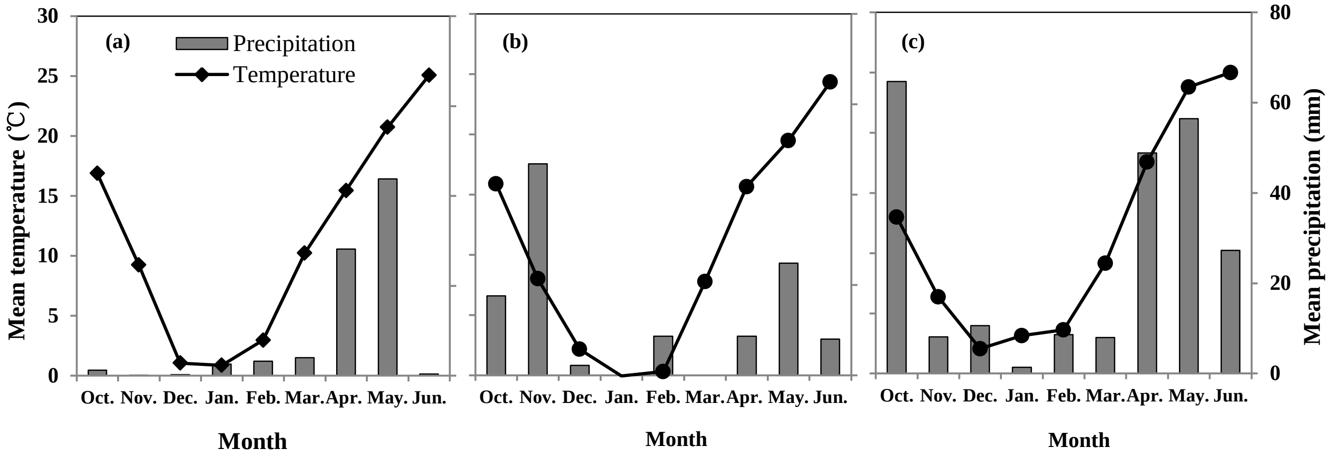

Supplementary Figure 1. Monthly precipitation and mean temperature in the three cropping years: 2014-2015 (a), 2015-2016 (b), and 2016-2017 (c)

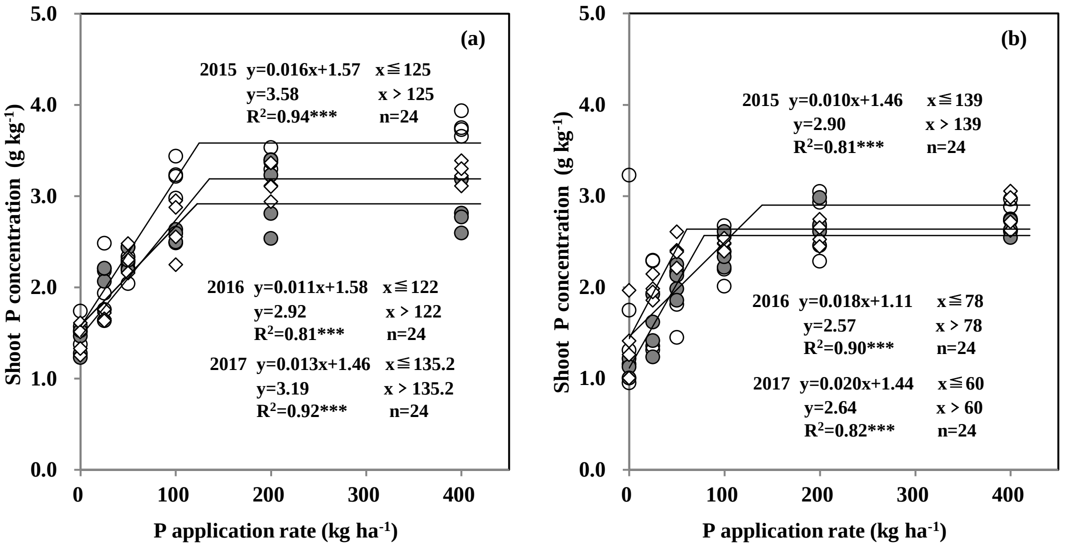

Supplementary Figure 2. P concentration in shoots of winter wheat at stem elongation (a) and anthesis (b) stages as affected by P application rate. Each data point is from one plot in 1 year. R^2 is the coefficient of determination of the segmented regression and the value behind "≤" or "＞" is the abscissa of the breakpoint of the relationship. *** indicates significant difference at $P<0.001$

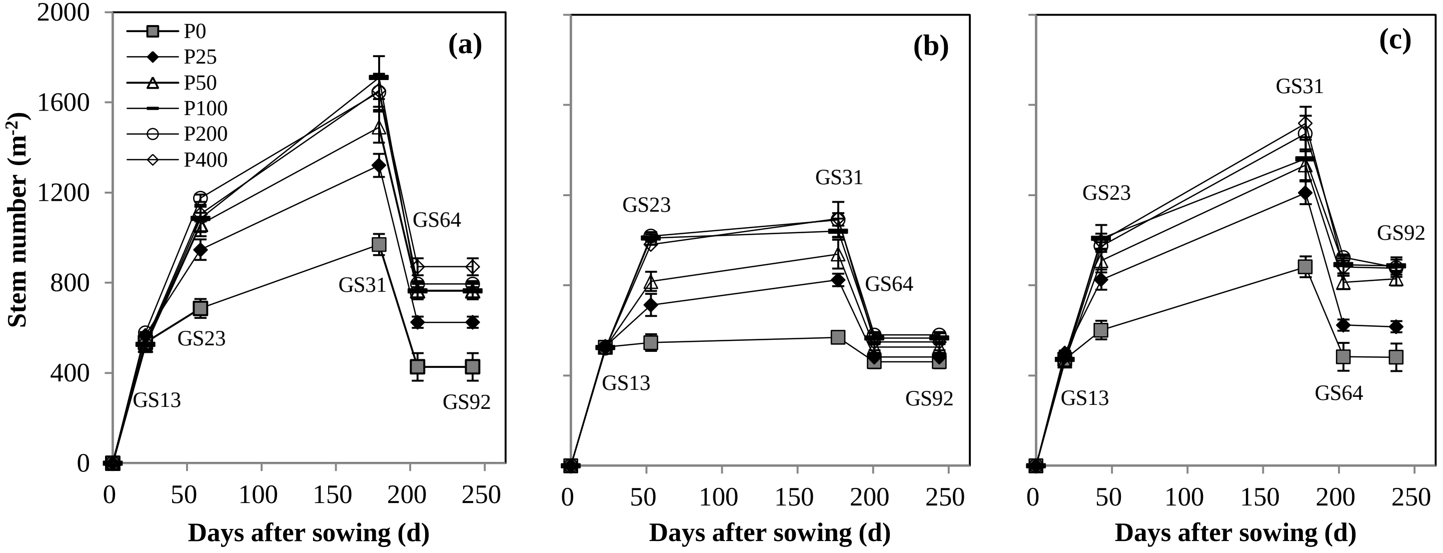

Supplementary Figure 3. Wheat stem number m^{-2} as affected by P application rate in 2015 (a), 2016 (b), and 2017 (c). Values are means ± S.E. of four replicates. GS13, GS23, GS31, GS64, and GS92 represent seedling, before winter, stem elongation, anthesis, and maturity stages of winter wheat, respectively

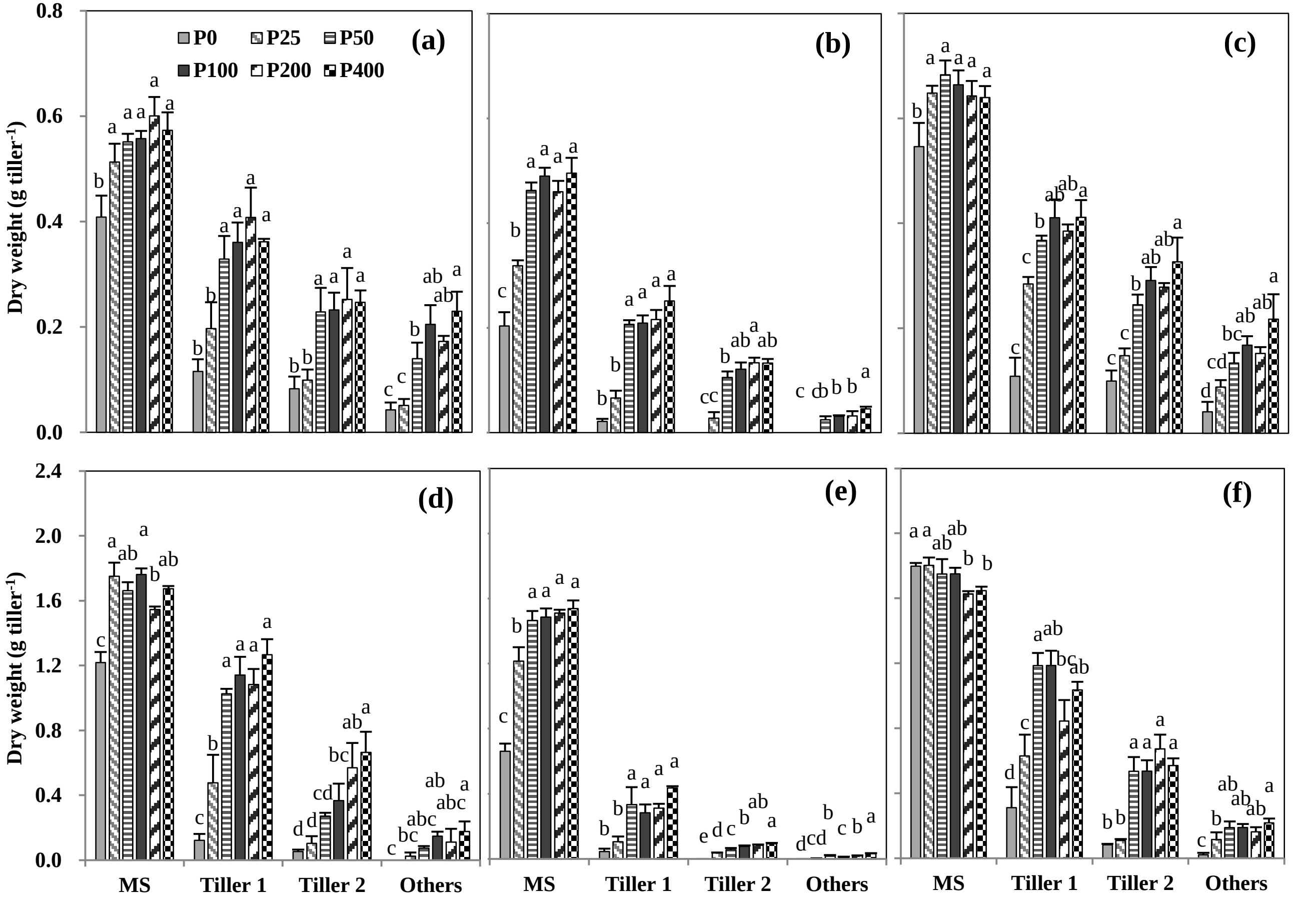

Supplementary Figure 4. Effects of P application rate on the dry weight of the main stem (MS), tiller 1, tiller 2, and other tillers at the stem elongation stage in 2015 (a), 2016 (b), and 2017 (c) and at anthesis in 2015 (d), 2016 (e), and 2017 (f). Values are means ± SE (n=4). For each kind of tiller, means with different letters are significantly different at P<0.05

Supplementary Table 1. Relationships between grain yield and yield components across P application rates and cropping years. TKW means thousand-kernel weight.

Independent variable	Correlation coefficients	Adjusted R^2	Path coefficients	Indirect path coefficients			
				Spike number	Grains $spike^{-1}$	TKW	Total
Spike number	0.71	0.50	0.77	-	0.12	-0.19	-0.06
Grains $spike^{-1}$	0.63	0.63	0.30	0.32	-	0.01	0.33
TKW	-0.25	0.66	0.27	-0.54	0.01	-	-0.52
